# Capturing foraging and resting behavior using nested multivariate Markov models in an air-breathing marine vertebrate

**DOI:** 10.1186/s40462-018-0134-4

**Published:** 2018-09-20

**Authors:** Ben G. Weinstein, Ladd Irvine, Ari S. Friedlaender

**Affiliations:** 1 Department of Fisheries and Wildlife, Marine Mammal Institute, Oregon State University, 2030 Marine Science Drive, Newport, OR 97365 USA; 2 Institute of Marine Sciences, Department of Ecology and Evolutionary Biology, UC Santa Cruz, 115 McAllister Way, Santa Cruz, CA 95060 USA

**Keywords:** Humpback whales, West Antarctic peninsula, Landscape ecology, Movement model, Hidden-markov models, Antarctic krill, Dive behavior

## Abstract

**Background:**

Matching animal movement with the behaviors that shape life history requires a rigorous connection between the observed patterns of space use and inferred behavioral states. As animal-borne dataloggers capture a greater diversity and frequency of three dimensional movements, we can increase the complexity of movement models describing animal behavior. One challenge in combining data streams is the different spatial and temporal frequency of observations. Nested movement models provide a flexible framework for gleaning data from long-duration, but temporally sparse, data sources.

**Results:**

Using a two-layer nested model, we combined geographic and vertical movement to infer traveling, foraging and resting behaviors of Humpback whales off the West Antarctic Peninsula. This approach refined previous work using only geographic data to delineate coarser behavioral states. Our results showed increased intensity in foraging activity in late season animals as the whales prepared to migrate north to tropical calving grounds. Our model also suggests strong diel variation in movement states, likely linked to daily changes in prey distribution.

**Conclusions:**

Using a combination of two-dimensional and three-dimensional movement data, we highlight the connection between whale movement and krill availability, as well as the complex spatial pattern of whale foraging in productive polar waters.

**Electronic supplementary material:**

The online version of this article (10.1186/s40462-018-0134-4) contains supplementary material, which is available to authorized users.

## Background

Animal movement provides insight into the ecology, life history and conservation needs of mobile species. An ongoing challenge in movement ecology is associating the spatial patterns of movement with the underlying behaviors such as foraging, predation, and reproduction [1–3]. Strengthening the connection between behavior and spatial patterns of movement requires additional mechanistic data beyond two dimensional representations of animal activity [4]. For marine animals, time-at-depth recorders are an important tool for creating more realistic models of foraging behaviors [5]. Yet incorporating such data with derived movement tracks is complicated by potential mismatches in the temporal frequency of data collection, as well as the ecological scale at which behaviors occur [2].

To create a more accurate depiction of behavior, analyses can move towards multi-layer models of nested behaviors [6]. Multi-layer models provide two advantages. First, datasets collected at different temporal and spatial scales can be analyzed jointly, without needing to down-sample data to a common observation frequency. For example, satellite-based geographic observations are often collected at relatively long intervals, compared to biometric [5] or accelerometer data [7]. Secondly, nested models allow greater partitioning among behavioral states. While many studies divide behavior into broad discrete states, we are often interested in finer behavioral categories that describe animal life history [8]. Rather than creating additional single-layer states, nested states allow us to first model the predictors of broad categories, such as foraging versus traveling, then subdivide these states based on local conditions or timeframes [2].

We use a nested multivariate movement model to capture both the two-dimensional space use and the vertical foraging behavior in Antarctic Humpback whales (*Megaptera novaeangliae)*. Antarctic Humpback whales are large filter feeding baleen whales commonly found in Antarctic waters during the austral summer. Humpbacks in these waters primarily feed on Antarctic krill (*Euphausia superba)*, which form large aggregative swarms during the austral summer and fall [9, 10]. At the broad scale, previous work has used two-dimensional visual observations to highlight areas of whale density [11–13], and satellite tag data to assess seasonal changes in movement [14]. Due to physiology and feeding mechanics, there is a minimal krill density at which foraging is energetically favorable for humpback whales, creating discrete bouts of feeding and traveling [15]. At broad scales, movement behaviors often last multiple days, as whales travel among and forage within productive patches of krill swarms [14]. However, single day tagging studies show a diversity of vertical behaviors related to foraging movements, with both resting and exploratory dives co-occurring with feeding dives within areas of high krill density [16]. Connecting these local patterns of dive intensity with regional patterns of movement is key in both delineating primary foraging habitat [17], as well as creating detailed physiological models of energetic demands [18].

Our aim is to create a broad-scale quantitative movement model that describes the spatial and temporal properties of dive behaviors, connects the high frequency observation of dives with the lower frequency of observation of geographic location, and realistically captures the time spent in foraging and resting behaviors at local scales. Weinstein and Friedlaender [14] divided movement into traveling and area-restricted search states, and showed an increase in the proportion of time in area-restricted search later in the austral fall. This could either come from 1) greater foraging intensity needed to fulfill energetic reserves before migration, 2) increased resting behavior to preserve energetic expenditure, or 3) reduced opportunity for traveling to new patches due to sea ice advance. By refining spatial patterns of movement with nested foraging behaviors, we can connect seasonal changes in behavior with potential changes in foraging intensity. The seasonal demands in energy expenditure may be critical in understanding the rebound in humpback populations from historic declines [19, 20], the effect of rapidly changing Antarctic marine environment [21, 22], and the potential competition with the Antarctic Krill fishery [11, 23].

## Methods

### Satellite tagging and tracking

We deployed 11 Wildlife Computers (Redmond, WA, USA) SPOT5 Platform Transmitting Terminals (PTTs) off the Western Antarctic Peninsula in 2016 (Table 1). Each tag is contained in a sterilized housing designed to penetrate the whale’s skin and blubber up to 290 mm, and is anchored in the tissue beneath the blubber with stainless steel barbs, with the transmitting antenna remaining free outside of the animal. All whales were presumed to be adults based on a minimum size of 12 m. Tags were deployed from a range of 3–10 m and placed near the dorsal fin, which contains the thickest blubber layer, and provides the greatest height to transmit positional information via the exposed antenna.

**Table 1:** Tag data for the 11 humpback whale tracks used in this study. The number of observed Argos locations and dive records are shown for each animal

Animal	Argos	Dive	Start	End
131,111	173	375	3/23/16 1:01	3/27/16 16:25
131,115	179	850	2/26/16 13:20	3/1/16 13:54
131,116	457	1475	2/26/16 21:28	3/6/16 14:18
131,127	2495	6780	4/26/16 14:05	7/13/16 14:01
131,128	52	60	4/26/16 13:25	4/27/16 6:36
131,130	151	1014	4/25/16 17:53	4/29/16 17:30
131,132	589	1703	4/25/16 20:23	5/10/16 14:11
131,133	2077	6419	4/26/16 13:24	7/5/16 16:04
131,134	653	1445	4/26/16 0:08	5/12/16 11:31
131,136	1970	4874	4/26/16 13:25	6/29/16 18:11
154,187	486	1380	3/21/16 20:05	4/2/16 16:10

Satellite transmissions were activated via a salt-water switch and received by the Argos satellite system which estimated the tag position based on the number and temporal distribution of transmissions received during a satellite pass. Tags sampled depth at 1 Hz using an onboard pressure sensor and we defined dives as submergence below 10 m for more than 20 s. Dive data were aggregated and transmitted in packages containing information on dive duration, maximum dive depth, and dive shape (determined by percentage of time spent at 80% of the maximum dive depth). All tags were set without duty cycling and attempted to transmit data on each surfacing. We filtered raw observations to remove transmissions without location data, duplicate timestamps, locations on land, and implausible speed between consecutive locations (20 km/hour) to create a conservative set of observations. We then filtered our observations to preserve continuous movement tracks, with a maximum 12-h window between points and a minimum track length of 24 h. Humpbacks engage in a northward migration from the West Antarctic Peninsula to western South America at the end of the austral fall. Migration events were identified as unidirectional movement north and removed from the analysis. The final dataset was comprised of 11 individuals, with 9283 Argos observations, and 26,375 recorded dives.

### Movement modeling

To associate spatial patterns of animal movement with predicted behavior phases, we begin with a hierarchical state-space model following [14, 24, 25]. This model allowed us to estimate multiple behavioral states, and account for measurement uncertainty using Markov Chain Monte Carlo (MCMC) simulation. As in previous work, we modeled whale movement as a function of the autocorrelation in step size and turning angles for two distinct behavioral phases: traveling and area-restricted search [24]. The traveling state is defined as large scale movements with high autocorrelation and small turning angles. Area-restricted search is defined as short step lengths with high turning angles.

#### Process model

1 $$ {\displaystyle \begin{array}{l}{Y}_{i,g,t+1}\sim \mathrm{Multivariate}\ \mathrm{Normal}\left({d}_{i,g,t},\sigma \right)\\ {}{d}_{i,g,t}={Y}_{i,g,t}+{\gamma_{s_{i,g,t}}}^{\ast }{T_{i,g,t}}^{\ast}\left(\ {Y}_{i,g,t}-{Y}_{i,g,t-1}\right)\\ {}{T}_{i,g,t}=\left[\begin{array}{cc}\cos \left({\theta}_{s_{i,g,t}}\right)& -\sin \left({\theta}_{s_{i,g,t}}\right)\\ {}\sin \left({\theta}_{s_{i,g,t}}\right)& \cos \left({\theta}_{s_{i,g,t}}\right)\end{array}\right]\\ {}S{\hbox{'}}_{i,g,t}\sim \mathrm{Multinomial}\left(\phi {\hbox{'}}_{i,g,t}\kern0.5em 1-\phi {\hbox{'}}_{i,g,t}\ \right)\\ {}\mathrm{logit}\left(\phi {\hbox{'}}_{i,g,t}\right)=\alpha {\hbox{'}}_{s{\hbox{'}}_{i,g,t},s{\hbox{'}}_{i,g,t-1}}\end{array}} $$

#### Observation model

2 $$ {\displaystyle \begin{array}{l}{w}_{i,g,t,u}\sim \mathrm{Multivariate}\ \mathrm{Normal}\ \left({\widehat{z}}_{i,g,t,u},{\tau}_{argos}\right)\\ {}\kern0.75em {\widehat{z}}_{i,g,t,u}=\left(1-{j}_{i,g,t,u}\right)\ast {Y}_{i,g,t-1}+{j_{i,g,t,u}}^{\ast }\ {Y}_{i,g,t}\end{array}} $$

This process model estimates the geographic location (*Y*) of individual (*i*) at time (*t*) along a track (*g*). This location is multivariate normally distributed with a mean location (*d*) and a variance in location (σ). The mean location is a first-order Markov process, such that it depends on the difference in location to the previous step (*Y*_*i*, *g*, *t*_ − *Y*_*i*, *g*, *t* − 1_), plus the movement at time *t*. This movement is a function of the degree of autocorrelation in step length (*γ*) and turning angles (T), with a mean turning angle (*θ*). Step lengths and turning angles are considered to come from two behavioral states, ‘traveling’ and ‘area-restricted search’. The predicted behavioral state (*S’*) is a Bernoulli draw with a probability of being in the traveling state (*ϕ*′_*i*, *g*, *t*_) or in the area-restricted search state (1 − *ϕ*′_*i*, *g*, *t*_).The prime notation denotes the first layer of behavioral states. The transition probabilities (*ϕ*^′^) among behavioral states (S′) depends on the current behavioral state and the behavioral state of the previous observation. To infer the sequence of behaviors, the most likely state (*ϕ*′_*i*, *g*, *t*_ > 0.5) is used for each observation.

The observation model describes our ability to detect location given the Argos observations. We chose to model movement on a six-hour time step, which was a conservative balance between accounting for gaps in Argos transmissions on data, and ensuring that behaviors do not change between observations. Within a time-step, individuals are assumed to move in a straight line, such that each Argos observation is expressed as a proportion of the six-hour time interval (*j*_*i*, *g*, *t*, *u*_). The variance in the Argos observation (*τ*_*argos*_) was fixed for each Argos error class following the results reported in Jonsen et al. (2005).

### Incorporating vertical movement

From the base model above, we added a nested behavioral state that describes the latent process that generates dive depths. The model is hierarchical, observations are first defined in terms of their geographic movement, and then refined based on vertical movement. We divided the area-restricted search state into foraging and resting sub-states based on single day tagging studies which showed both behaviors occurring during foraging movements [16]. We chose not to further partition the traveling state, since our aim is to clarify the behaviors associated with the area-restricted search state. The behavioral sub-state (S”) is a separate Markov chain that generates observed dive depths a mixture of normally distributed dive distributions.

3 $$ {\displaystyle \begin{array}{l}{S}_{i,g,t,u}^{\hbox{'}\hbox{'}}\sim \mathrm{Multinomial}\left({\phi}_{i,g,t,u}^{\hbox{'}\hbox{'}}\kern0.5em 1-{\phi}_{i,g,t,u}^{\hbox{'}\hbox{'}}\ \right)\\ {}\mathrm{logit}\left({\phi}_{i,g,t,u}^{\hbox{'}\hbox{'}}\right)={\alpha^{\hbox{'}\hbox{'}}}_{s_{i,g,t}^{\hbox{'}},{s^{\hbox{'}\hbox{'}}}_{i,g,t,u-1}}\\ {}{Dive}_{i,g,t,u}\sim Normal\left({\mu}_{S_{i,g,t,u}^{\hbox{'}\hbox{'}}},{\sigma}_{S_{i,g,t,u}^{\hbox{'}\hbox{'}}}\right)\end{array}} $$

Note that transition probability relates both to the sub-state of the previous dive (S”), as well as the behavioral state of the top layer (S’). We arrived at this model through extensive exploration of single layer models which could not balance the multivariate information of both 2-dimensional and 3-dimenisonal data without needing unrealistic prior bounds on the latent behavioral states.

Dive priors were sampled from a zero-truncated normal distribution since dive depths must be positive. The traveling state was given a broad dive prior with a mean dive depth between 0 and 30 m. Animals are unlikely to dive deeper than 100 m during traveling phases, as deeper dives would decrease horizontal speed and increase physiological demand. Resting dives were similarly constrained to be a mean depth of 0 to 30 m [16]. Based on evaluation of the data and previous dive profiles, foraging dives were given a broad prior, with mean depth between 50 m and 250 m [26, 27]. We believe these priors are biologically defensible, reasonably broad, and promote convergence during Bayesian simulation. Simulations were run in R (R Development Core Team 2015) and JAGS [28] with 2 MCMC chains running in parallel, each running for a total of 30,000 iterations. The first 28,000 iterations were discarded as burn-in. The final 2000 iterations were thinned by 4 to reduce autocorrelation and the computational burden of saving a large number of latent behavioral states to file. Chain convergence was assessed by comparing the within chain variances to the between chain variance using the Gelman-Rubin statistic. Parameters with Gelman-Rubin ($\widehat{r}\Big)$ of less than 1.1 were considered converged following Gelman and Hill [29]. Source code and data visualizations are available on github (https://bw4sz.github.io/WhalePhys/).

## Results

**Fig. 1:**
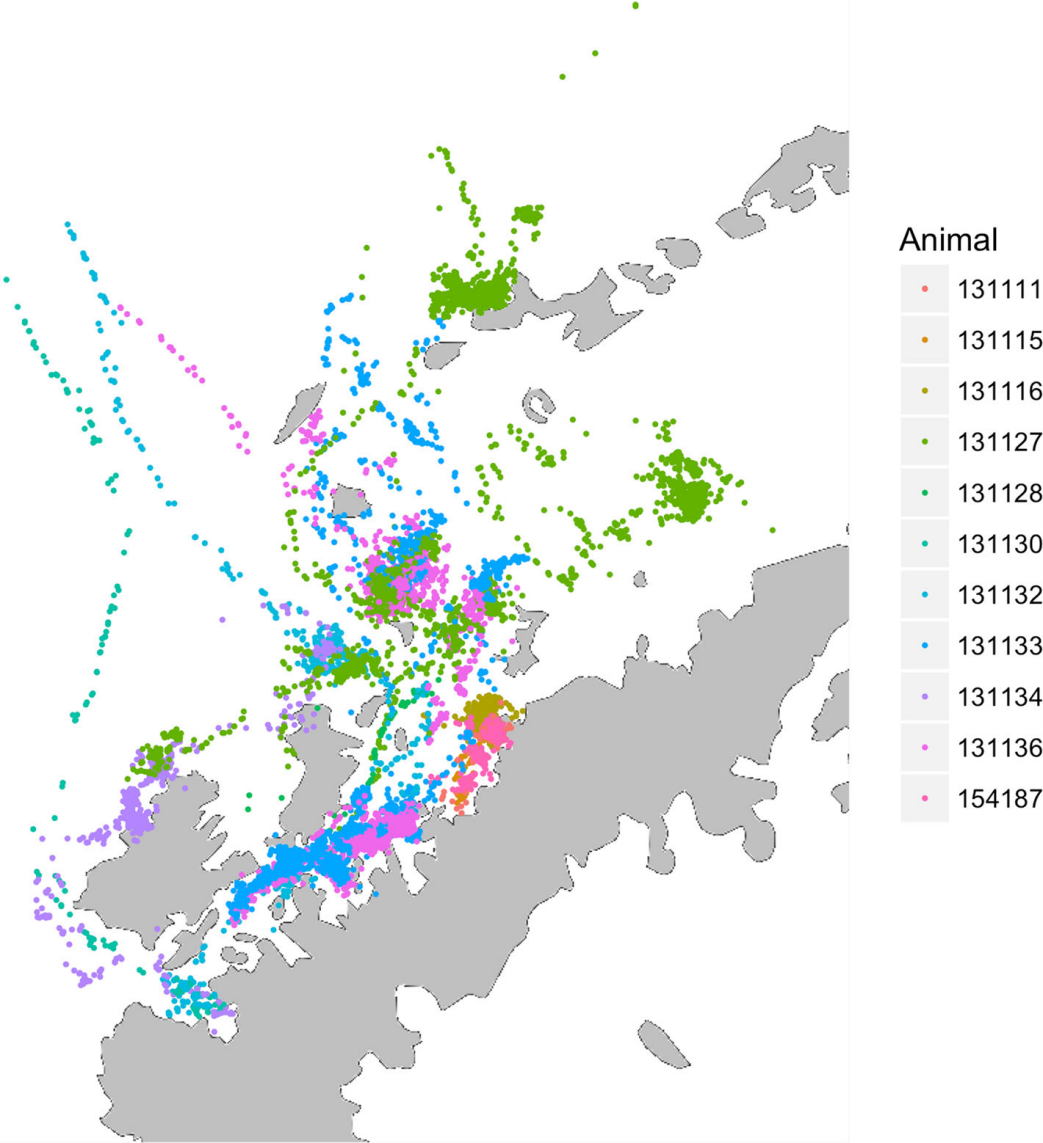
Filtered geographic observations of 11 adult humpback whales tracked by satellite tag in waters off the West Antarctic Peninsula during Feb-May 2016

**Fig. 2:**
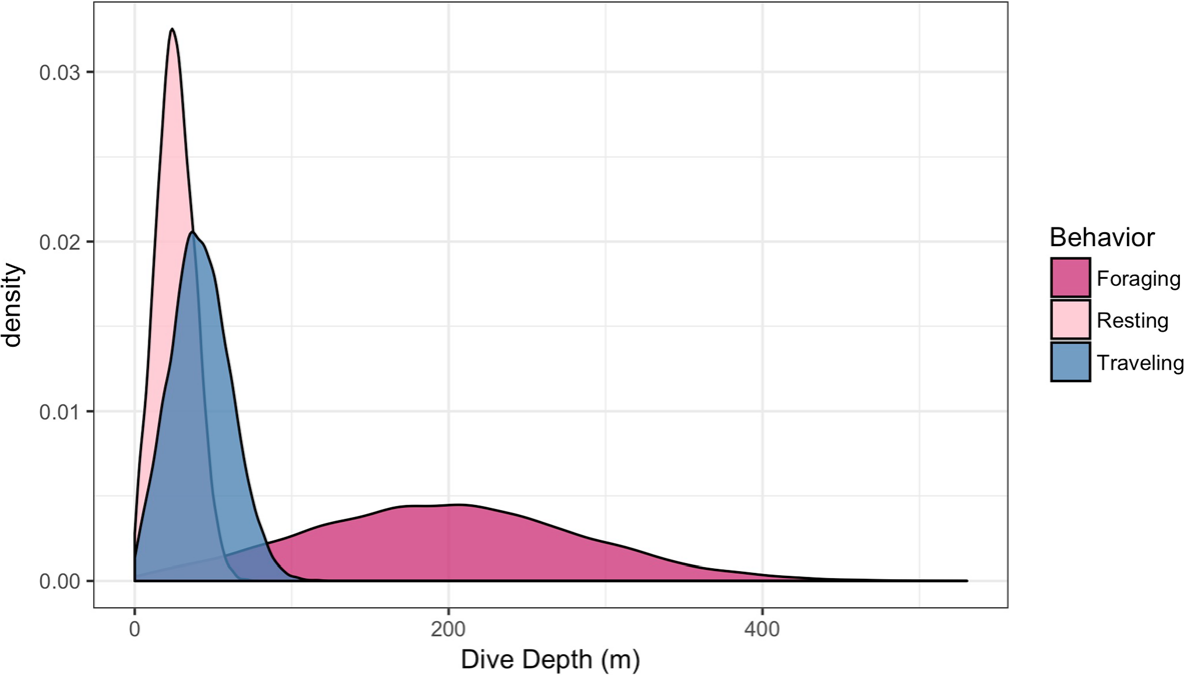
Predicted dive depth distributions of satellite tagged humpback whales for each of three latent movement behaviors predicted by a nested multivariate movement model

**Fig. 3:**
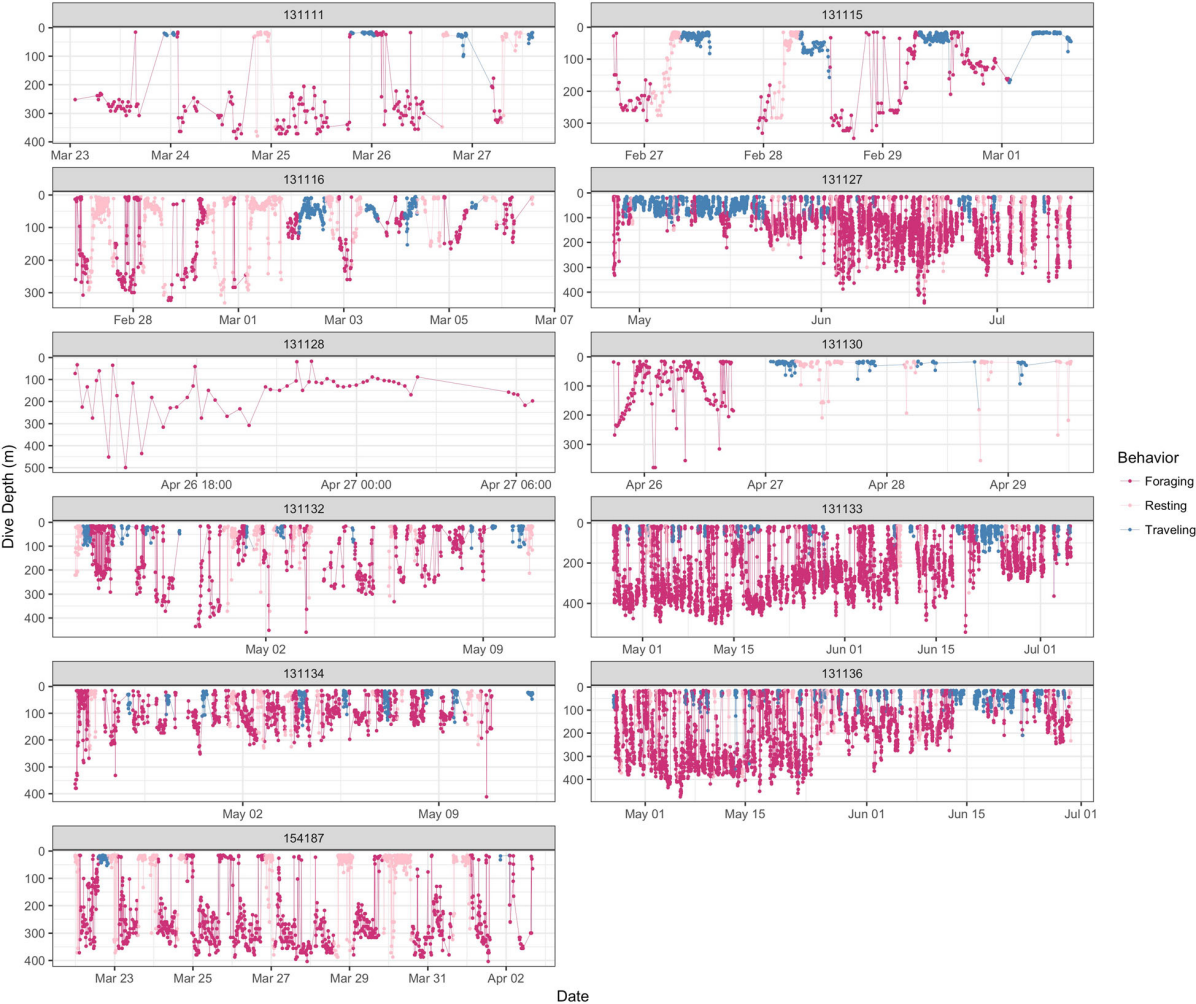
Dive profiles for 11 satellite-tagged humpback whales off the West Antarctic Peninsula from Feb-May 2016. Inferred behavioral state for each dive is based on a nested movement model in which foraging and resting states are nested within the area-restricted search state. Tracks were combined for each animal to show a single sequence of inferred behaviors

**Fig. 4:**
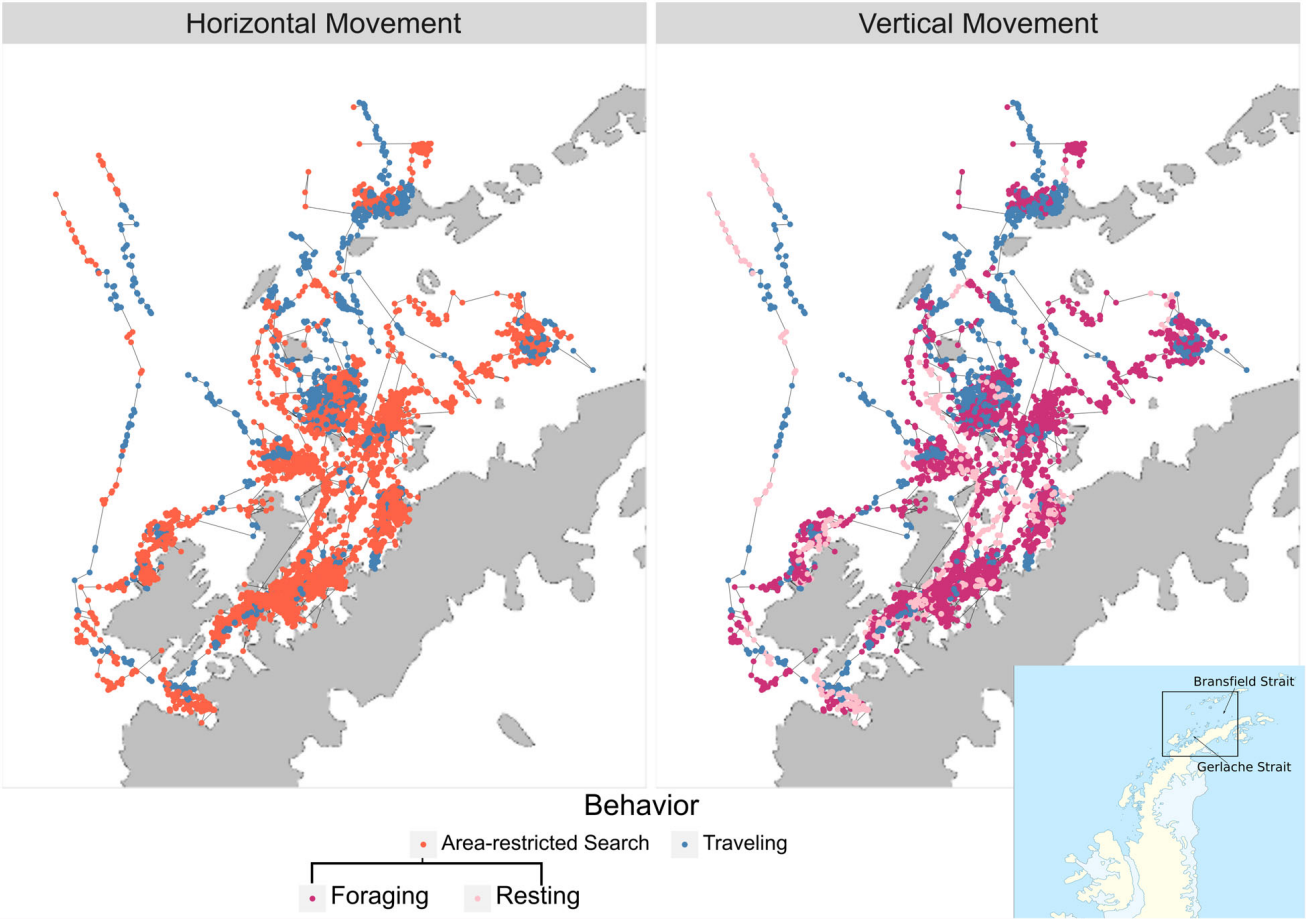
Spatial prediction of the behavioral states for 11 satellite tagged humpback whales off the West Antarctic Peninsula from Feb-May 2016. The observed locations are first partitioned into traveling and area-restricted search states based on the two-dimensional pattern of horizontal movement. The area-restricted search states are then subdivided into foraging and resting sub-states based on vertical dive depths. We did not subdivide the traveling state based on dive depths, therefore the traveling state appears in both maps

Our filtered data contained 8958 Argos observations (Fig. 1) and 21,417 dive records for 11 adult humpback whales. The average number of dives per individual was 1947, with an average deployment duration of 26.71 days. The movement model converged in 13.5 h with Rhat statistics less than 1.1 for all parameters [29]. Movement parameter estimates can be found in Additional file 1: Table S1, and the estimated latent dive distributions for each behavior are shown in Fig. 2. The area-restricted search state showed strong temporal autocorrelation (${\alpha}_{ARS, ARS}^{\prime }$ = (0.82,0.88)), with high autocorrelation both in the foraging (${\alpha}_{Foraging, Foraging}^{\prime \prime }$ = (0.95,0.96)) and resting state (${\alpha}_{Resting, Resting}^{\prime \prime }$ = (0.87,0.89)) (Figs. 2 and 3). The spatial pattern of traveling and area-restricted search states largely matched previous work in [11, 14], with the majority of area-restricted search events in the Gerlache and Bransfield straits (Fig. 4). Within area-restricted search, the mean percentage of time spent in foraging state (mean = 58.2%, sd = 0.23) was greater than the mean percentage of time spent in the resting state (mean = 22.0%, sd = 0.15). Traveling dives had an estimated mean depth of 41.0 m (40.5 m, 41.5 m) with the majority of observed dives between 21.5 m (25th quantile) and 58.5 m (75th quantile). Foraging dives had an estimated mean depth of 194.3 m (192.6 m, 195.9 m) with the deepest observed dive of 543.5 m. The majority of observed foraging dives were between 88.5 m (25th quantile) and 267.5 m (75th quantile). Resting dives had an estimated mean depth of 25.1 m (25.1 m, 25.9 m) with the majority of observed dives between 18.5 m (25th quantile) and 49.0 m (75th quantile). Resting states occasionally included single deep dives (max = 395.5 m) followed by a return to typical short dives.

**Fig. 5:**
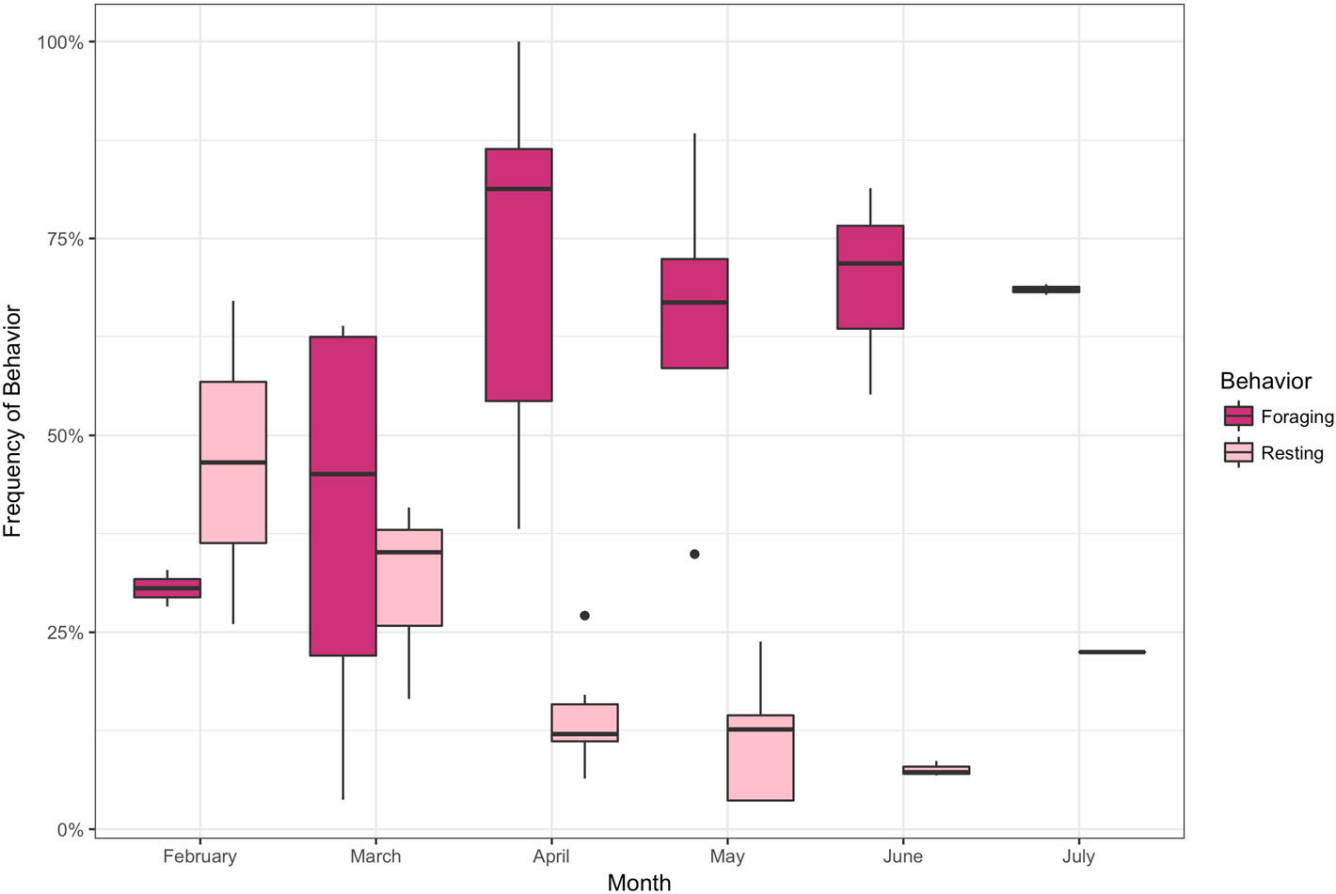
Seasonal frequency of foraging and resting states for humpback whales satellite tagged off the West Antarctic Peninsula inferred using a nested multivariate movement model. The traveling behavior did not show a consistent seasonal pattern and is not shown below

**Fig. 6:**
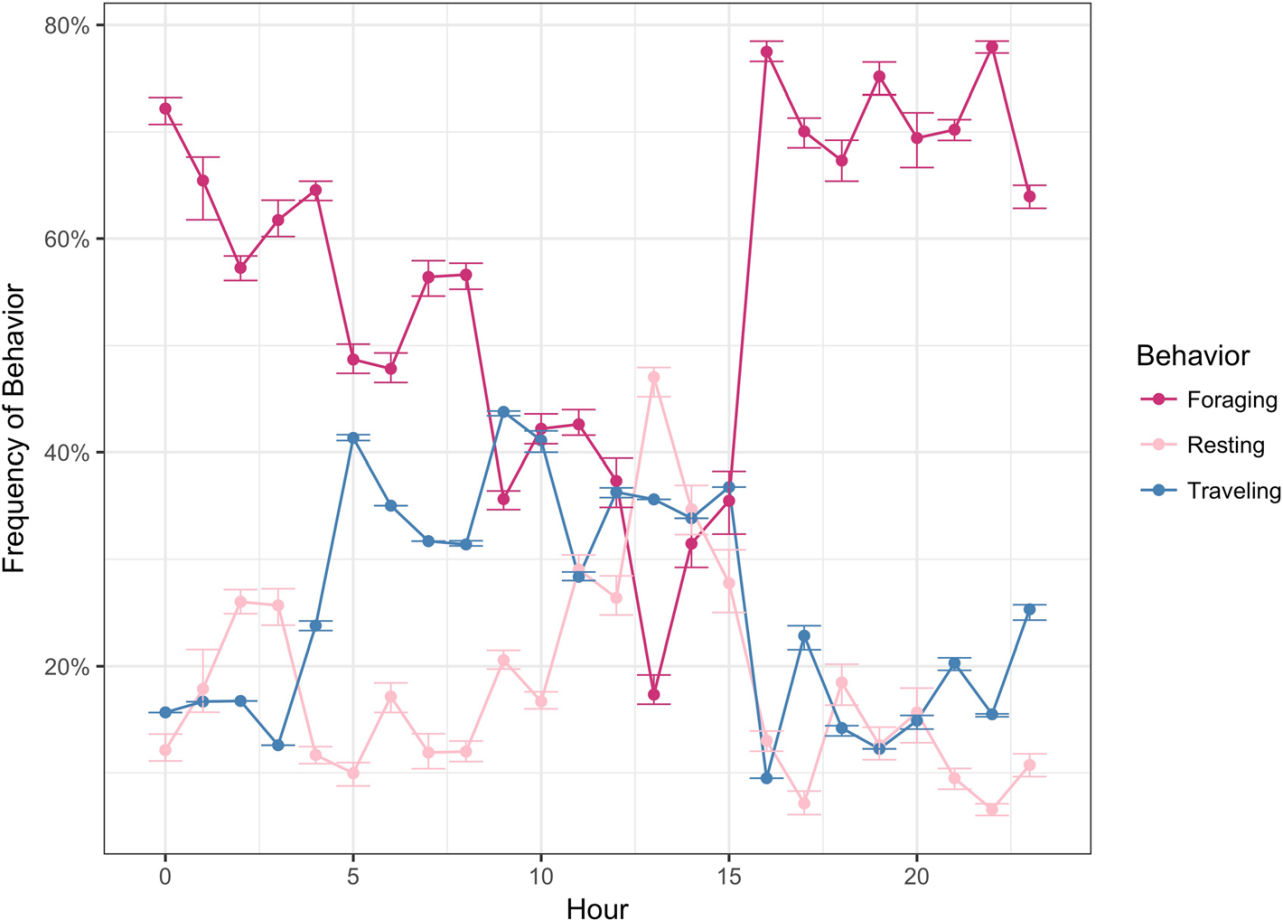
Daily behavior patterns of satellite tagged humpback whales off the West Antarctic Peninsula inferred using a nested multivariate movement model. The total frequency of behavior across all individuals shows a diurnal drop in foraging behavior during daylight hours. Error bars show the uncertainty in estimates of the behavior states for 50 draws of the posterior distribution for each observation

Partitioning the results by time, the model shows a seasonal increase in the foraging state with the mean foraging frequency rising to nearly 70% in May–June (Fig. 5). While there is not a clear seasonal pattern in dive depths, one noticeable finding is the frequency of late season deep dives in May and June with 616 observed dives greater than 400 m. Given the uneven sampling throughout the year, we avoid overly interpreting this finding, but believe it is worth further investigation. Finally, partitioning dive behavior by time of day showed a decrease in the frequency of foraging state between 10 am-3 pm (Fig. 6).

## Discussion

Using a nested multivariate movement model, we combined two-dimensional movement data with vertical dive data to model foraging behavior in adult humpback whales off the West Antarctic Peninsula. Our foremost goal was to investigate the seasonal increase in area-restricted search state, reported in [14], by explicitly incorporating dive behavior as a more refined signature of whale behavior. By partitioning area-restricted search into foraging and resting states based on an animal’s vertical movement, we find a strong seasonal increase in foraging dive behavior in the late austral fall. This observed increase in foraging frequency coincides with changes in krill abundance and distribution in the coastal and continental shelf waters around the Antarctic Peninsula. The life-history of krill in the West Antarctic Peninsula remains poorly understood, but the general pattern is adult krill feeding along the continental shelf in the austral summer, followed by an inshore migration towards ice-covered areas during the austral fall [10, 30]. As sea-ice forms, krill aggregate in larger swarms in coastal bays [23, 31]. Humpbacks mirror these changes in distribution, with large aggregations of individuals nearshore and along seasonal ice edges [9, 11, 17].

The response of whales to the change in krill distribution may represent a strategy to increase fat reserves prior to migration [32]. The greater density of krill may create more efficient foraging opportunities, by reducing the number of dives needed to sustain energetic demands. If foraging is indeed more productive in the late season, this creates a tradeoff between leaving for the calving grounds in order to fulfill reproductive needs, and staying in the Antarctic to increase fat reserves. While the presence of seasonal sea-ice prevents animals from staying year-round, we expect immature or non-reproductively active whales to stay later in the season due to reduced pressure on the timing of northward migration. As the average number of ice free days along the West Antarctic Peninsula rises [33], the ability for whales to remain longer in Antarctic waters may affect the recovery of whales from historic harvest [34], create competition with other krill predators [35], and potential conflict with the growing krill fishery [23, 36, 37].

The strong connection between whale and krill distribution also included daily vertical movements [10]. Showed elevated nighttime concentrations of krill near the surface followed by descent to greater than 200 m during the day in West Antarctic waters. Short-term observations of both whales and krill suggested that whales actively explore the water column to assess the vertical distribution of prey [16]. Our results showed an increase in the frequency of foraging behavior at night. This pattern of behavior also helps explain the presence of isolated deep dives within the resting state, since deep dives are likely exploratory behaviors to assess krill presence in the water column. Deep dives are energetically expensive, and the diel behavior suggest individuals are balancing the cost of dive expenditure and foraging efficiency. Without the temporal context of the resting state, exploratory deep dives may be mistakenly labeled as foraging events. These observations are symptomatic of the larger challenge of matching individual observations with complex latent behavior over discrete time [38].

Detailed examination of our results underscores the need for further development of movement models to represent latent behavior as a combination of local movement and regional context. For example, we observed a potential foraging bout north of Livingston Island that was classified as traveling because individual 131,127 performed a series of short linear movements, with shallow dives to 80-100 m over a multiple day period. The consistency and tight spatial area of this movement suggests this is likely a foraging event on a shallow krill swarm near the continental shelf. In addition, the location of this event matches an observed krill hotspot reported by [39]. The model may have failed to recognize this foraging bout because it is the only foraging activity along the continental shelf in our dataset. It is possible that the foraging behavior in this area is distinct from deeper diving events in the Gerlache and Bransfield straits, which make up the majority of our dataset. Similarly, observations of individuals migrating northward across the Drake Passage were likely mislabeled resting based on more erratic movements. Given that the majority of our movement data is within more protected inshore waters, navigating the rougher seas of the open ocean may lead to distinct traveling behaviors. The challenge of collecting movement data across broad spatial scales has limited the development of flexible behavioral models that vary as a function of regional distribution. By combining data across scales, we can better match observed movement patterns with a reasonable number of latent behavioral states, rather than adding additional behavioral states for movement in each new regional context.

A natural extension of the nested behavioral model is the incorporation of environmental covariates for the transitions among states and sub-states [25]. By combining multiple behavioral layers, these approaches could allow ecologists to test the spatial and temporal scale at which environmental covariates become informative. While this holds great promise in moving towards a mechanistic model of animal movement [2], we believe it will be difficult to apply to Antarctic humpbacks foraging on krill. While broad scale predictors such as bathymetry and distance to shore are correlated with humpback presence [17], our exploratory analysis shows relatively little explanatory power on specific behavioral states. Our results, and prior work, show that whale foraging is tightly coupled with krill presence and density [9, 40]. While there has been significant effort to create environmental models of krill abundance [13, 41], a recent review highlighted a lack of predictive power at the local scale [42]. Beyond the tendency of juvenile krill to aggregate near the seasonal ice edges [43], the seasonal and daily vertical movements of krill relate to a complex combination of oceanographic, productivity, and tidal forces [44]. Given the lack of these fine-scale data needed to predict krill aggregations, direct modeling of krill-predator dive behavior may be a more tractable way of assessing the areas of potential conservation priority in Antarctic waters [13, 36].

An intriguing possibility of nested behavioral models is the power to explore the temporal and spatial scale of movement behaviors. By modifying the step duration at each behavioral layer, it will be possible to evaluate the temporal persistence in behaviors, and spatial predictors that best match different temporal scales [38]. For example, at fine scales (100 s of meters), whales are responding to changes in resource abundance, whereas at the multiple kilometer scale, humpbacks must avoid encroaching sea-ice to ensure consistent access to the surface to breath. Currently, the framework that describe species occurrence is analytically separate from models that describe movement behaviors. Combining these two fields through nested models, we can begin to ask what are the predictors of animal behavior conditional on species occurrence and broad scale patterns of movement.

## Conclusions

As biologger sensors become more advanced, ecologists are challenged to combine the data collected at different temporal and spatial extents to infer animal behavior. The spatial movements of whales are captured at a lower temporal frequency than the dive movements, but both encode information on foraging behavior. Our combined model can more realistically model foraging behavior by explicitly including vertical movement as a predictor of a nested latent state. Our results show a strong increase in foraging frequency in the late austral fall, coinciding with published patterns of krill movement. Given the rapid environmental change in the region, our study will help outline the physiological demands and the potential changes in whale distribution during global change in polar waters.

## Additional file

Additional file 1: **Table S1.** Parameter estimates for the humpback foraging, traveling, and resting behavioral states inferred from the nested multivariate movement model. (DOCX 19 kb)

## Abbreviations

ARS: Area-restricted search
