# Supplementary material for: Capturing foraging and resting behavior using nested multivariate Markov models in an air-breathing marine vertebrate
Source: Mov Ecol. 2018 Sep 20;6:16. doi: 10.1186/s40462-018-0134-4 (PMC6146519; doi:10.1186/s40462-018-0134-4)
Supplement: Supplementary file 1 — Table S1. Parameter estimates for the humpback foraging, traveling, and resting behavioral states inferred from the nested multivariate movement model. (DOCX 19 kb) [file 40462_2018_134_MOESM1_ESM.docx]

# Supplemental Figures

Table S1 Parameter estimates for the humpback foraging, traveling, and resting behavioral states inferred from the nested multivariate movement model.

| Parameter | Description | mean | Lower 5^th^ | Upper 95^th^ |
| --- | --- | --- | --- | --- |
| $\alpha_{ARS}^{'}$ | Probability of remaining traveling when traveling at previous time step | 0.51 | 0.45 | 0.57 |
| $\alpha_{Traveling}^{'}$ | Probability of transitioning to travel when in ARS at previous time step | 0.15 | 0.13 | 0.18 |
| $\mu_{Traveling}$ | Mean dive depth (m) for traveling state | 40.94 | 40.47 | 41.46 |
| $\mu_{Foraging}$ | Mean dive depth (m) for foraging substate | 194.32 | 192.65 | 195.92 |
| $\mu_{Resting}$ | Mean dive depth (m) for resting substate | 25.51 | 25.12 | 25.92 |
| $\gamma_{Traveling}$ | Movement autocorrelation when traveling | 0.53 | 0.33 | 0.75 |
| $\gamma_{ARS}$ | Movement autocorrelation when in ARS | 0.21 | 0.09 | 0.33 |
| $\alpha_{Foraging}^{''}$ | Probability of remaining in foraging when foraging at previous time step | 0.96 | 0.95 | 0.96 |
| $\alpha_{Resting}^{''}$ | Probability of transitioning to foraging when resting in previous time step | 0.12 | 0.11 | 0.13 |
